# Ca^2+^ signaling in arterioles and small arteries of conscious, restrained, optical biosensor mice

**DOI:** 10.3389/fphys.2014.00387

**Published:** 2014-10-07

**Authors:** Seth T. Fairfax, Joseph R. H. Mauban, Scarlett Hao, Mark A. Rizzo, Jin Zhang, W. Gil Wier

**Affiliations:** Department of Physiology, University of Maryland School of MedicineBaltimore, MD, USA

**Keywords:** two-photon, arteriole, Calcium, imaging, sympathetic nervous system

## Abstract

Two-photon fluorescence microscopy and conscious, restrained optical biosensor mice were used to study smooth muscle Ca^2+^ signaling in ear arterioles. Conscious mice were used in order to preserve normal mean arterial blood pressure (MAP) and sympathetic nerve activity (SNA). ExMLCK mice, which express a genetically-encoded smooth muscle-specific FRET-based Ca^2+^ indicator, were equipped with blood pressure telemetry and immobilized for imaging. MAP was 101 ± 4 mmHg in conscious restrained mice, similar to the freely mobile state (107 ± 3 mmHg). Oscillatory vasomotion or irregular contractions were observed in most arterioles (71%), with the greatest oscillatory frequency observed at 0.25 s^−1^. In a typical arteriole with an average diameter of ~35 μm, oscillatory vasomotion of a 5–6 μm magnitude was accompanied by nearly uniform [Ca^2+^] oscillations from ~0.1 to 0.5 μM, with maximum [Ca^2+^] occurring immediately before the rapid decrease in diameter. Very rapid, spatially uniform “Ca^2+^ flashes” were also observed but not asynchronous propagating Ca^2+^ waves. In contrast, vasomotion and dynamic Ca^2+^ signals were rarely observed in ear arterioles of anesthetized exMLCK biosensor mice. Hexamethonium (30 μg/g BW, i.p.) caused a fall in MAP to 74 ± 4 mmHg, arteriolar vasodilation, and abolition of vasomotion and synchronous Ca^2+^ transients.

**Summary**: MAP and heart rate (HR) were normal during high-resolution Ca^2+^ imaging of conscious, restrained mice. SNA induced continuous vasomotion and irregular vasoconstrictions via spatially uniform Ca^2+^ signaling within the arterial wall. FRET-based biosensor mice and two-photon imaging provided the first measurements of [Ca^2+^] in vascular smooth muscle cells in arterioles of conscious animals.

## Introduction

Here we report the first non-invasive measurements of intracellular smooth muscle [Ca^2+^] in conscious mouse arterioles. Two major considerations motivated this work: (1) Ca^2+^ signaling in arteries under physiological conditions with normal arterial blood pressure and intrinsic regulation via circulating hormones and the autonomic nervous system had never before been observed. (2) Studies of Ca^2+^ signaling in experimental hypertension have been complicated by the effects of anesthesia to reduce arterial blood pressure probably by reducing sympathetic nerve activity (SNA) to arteries. As a consequence, genuinely representative *in vivo* measurements of intracellular [Ca^2+^] did not exist.

**Figure 1:**
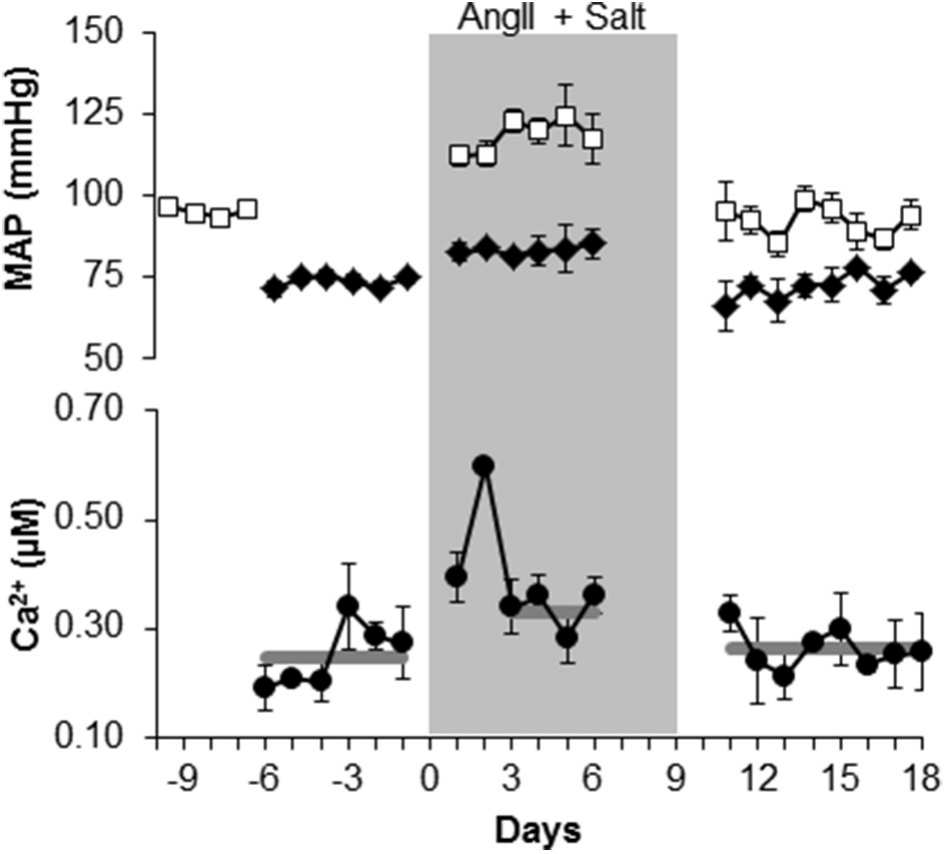
**Mean arterial pressure (MAP) and smooth muscle [Ca^2+^] increase in arterioles of exMLCK mice during chronic infusion of Angiotensin II and a high salt diet**. MAP (top panel) in conscious state (open squares) and in anesthetized state (closed diamonds), during simultaneous measurements of [Ca^2+^] (lower panel; *n* = 5 animals). For [Ca^2+^] measurements, each point represents a mean ± s.e.m of 2 or 3 mice, because mice were imaged in 2 groups on alternating days. Missing data are from days in which imaging or MAP measurements were not performed. Gray lines indicate the mean values over the corresponding time periods. MAP increased by 26 mmHg during AngII/salt (shaded area) when animals were conscious and by 11 mmHg when anesthetized. During the last 4 days of the AngII/salt period, mean [Ca^2+^] was increased by 85 nM. Removal of AngII/salt reduced both MAP and arteriolar [Ca^2+^] (reproduced with Permission from AJP, Heart and Circulatory Physiology).

We have previously reported non-invasive, serial measurements of [Ca^2+^] in arterioles of intact ears of anesthetized hypertensive mice (Mauban et al., 2014). In those studies, anesthesia was used for the sole purpose of immobilizing the animal for microscopic imaging, as no discomfort or pain was associated with imaging. Particular blood vessels were studied repeatedly over weeks, enabling “longitudinal” measurements of [Ca^2+^], with an accompanying increase in statistical power compared to “cross-sectional” studies of different animals at different times. Cytoplasmic [Ca^2+^] was observed to be slightly elevated during a period of hypertension induced by chronic administration of Angiotensin II and a high salt diet (Figure 1). Nevertheless, mean arterial pressure (MAP) was significantly reduced during the periods in which arterial [Ca^2+^] was measured, and this decrease was clearly due to the anesthesia. Anesthesia likely reduced SNA to arteries (Seagard et al., 1984) as well as possibly increasing activity of the renin-angiotensin system (Ullman et al., 2003). This is a particularly important problem because Angiotensin II (acting in the brain) and enhanced SNA are thought to be a major cause of increased vascular resistance in hypertension (Blaustein et al., 2012; Parati and Esler, 2012). Thus, it remains to be determined what is the arterial [Ca^2+^] and what transient changes in arterial Ca^2+^ activate contractions when MAP and SNA are not depressed by anesthesia, both in the normal basal state of the animal and in experimental models of disease.

Several developments in technology were key to the success of earlier studies, and also to the present work: (1) the use of transgenic optical biosensor mice, that express the genetically encoded FRET-based Ca^2+^ indicator molecule, exMLCK, specifically in smooth muscle (Isotani et al., 2004; Wier et al., 2008; Raina et al., 2009), (2) two-photon fluorescence microscopy, which permits non-invasive optical imaging within intact tissue, and (3) use of implanted telemetric blood pressure transducers for non-invasive measurement of arterial pressure in conscious animals. Importantly, none of these techniques involve pain or discomfort to the animal, enabling similar studies to be performed in conscious mice, provided that the mouse can be sufficiently motionless for imaging. Hence in the present study utilized techniques of restraint for conscious mice, developed previously for studies of neuronal activity (e.g., Guo et al., 2014) or blood flow (Drew et al., 2011) in cerebral vessels of conscious mice equipped with optical cranial windows or thinned skulls (Shih et al., 2012).

One anesthetic cocktail involving a mixture of ketamine, medetomidine, and atropine is able to prevent the reduction of MAP typically observed in anesthetized mice; however it strongly reduces heart rate (HR) (Zuurbier et al., 2014). Maintenance of MAP with such an anesthetic mixture is desirable to support the animal during surgery, but because it likely increases vascular resistance, it is not acceptable for study of the underlying mechanisms of vascular function. Consequently, conscious restraint as developed previously for studying neuronal activity in awake mice (Shih et al., 2012) is the necessary methodology to avoid disturbance of the natural cardiovascular system. Still, an earlier report (Gross and Luft, 2003) indicated that a generalized stress response might elevate MAP in mice under physical restraint. Thus, to ensure that these findings were reproducible for all physical restriction models, we began the present study by examining the effects of conscious restraint on MAP in our custom-made apparatus designed for ear imaging.

The methodology of Ca^2+^ measurements by two-photon excitation of exMLCK has been presented in detail previously (Mauban et al., 2013, 2014). Briefly, the kinetics and affinity of exMLCK for Ca^2+^ permit the detection of asynchronous propagating Ca^2+^ waves (Miriel et al., 1999) and synchronous Ca^2+^ oscillations (Mauban et al., 2001), but are insufficient to measure small localized Ca^2+^ transients, such as smooth muscle Ca^2+^ sparks (Nelson et al., 1995; Miriel et al., 1999) or junctional Ca^2+^ transients (jCaTs) (Lamont and Wier, 2002). ExMLCK is also a ratiometric FRET-type Ca^2+^ indicator, which allows for the quantification of steady-state [Ca^2+^] as well as [Ca^2+^] changes over a few hundred milliseconds. The FRET indicator also ameliorates complications arising from tissue motion and heterogeneities in expression of exMLCK among different cells (Zhang et al., 2010). Finally, exMLCK is well-suited to quantifying the concentration and transient activities of Ca^2+^ that activate contraction, because the binding affinity of exMLCK to Ca^2+^ is very similar to that of the endogenous MLCK (Hong et al., 2009; Wang et al., 2013).

We employed these methods and technologies to make the first observations of non-invasive, intracellular smooth muscle [Ca^2+^] in arterioles of conscious mice. All of the data obtained were acquired while the mice were awake, still and exhibited normal MAP. We present the intracellular [Ca^2+^] during quiescence, spontaneous transient Ca^2+^ signals, and during vasomotion. Lastly, we confirmed the actions of SNA within the ear vasculature.

## Materials and methods

### Animals

All experiments were approved by the Institutional Animal Care and Use Committee of the University of Maryland School of Medicine. The transgenic mouse line (exMLCK), which expresses a FRET-based Ca^2+^/MLCK biosensor (Isotani et al., 2004), was the same as used previously (Mauban et al., 2014). All mice were maintained on 12:12-h light/dark schedule at 22–25°C and 45–65% humidity and fed *ad libitum* on a standard rodent diet and tap water. Mice were 12–20 weeks old.

### Telemetric recording of arterial blood pressure

Arterial blood pressure was recorded via telemetric pressure transducers (TA11PA-C10, Data Science International, Minneapolis, MN, USA) implanted in the right carotid artery. A period of recovery lasting 7–10 days was provided before animals were utilized for experiments.

### Installation of head restraints

Mice were anesthetized using 2% isoflurane in 100% oxygen. The scalp was trimmed using hair clippers, cleaned with iodine and alcohol, and the dorsal skull surface was removed with surgical scissors. Sterile cotton swabs were used to scrape away the periosteum, clean and dry the skull surface. A 2.5 cm long threaded stainless steel bar was secured in a horizontal position above the skull, extending toward the non-experimental ear using 3M ESPE dental cement (Dentsply). Animals recovered for 3 days before experimentation. During experiments, the animals' heads were fixed in position by attaching the threaded bar to an anchored stage, their bodies were immobilized inside a custom-made conical tube, and their depilated ears were positioned on a flat horizontal silicone platform as previously detailed (Mauban et al., 2014).

### Imaging the mouse ear

**Figure 2:**
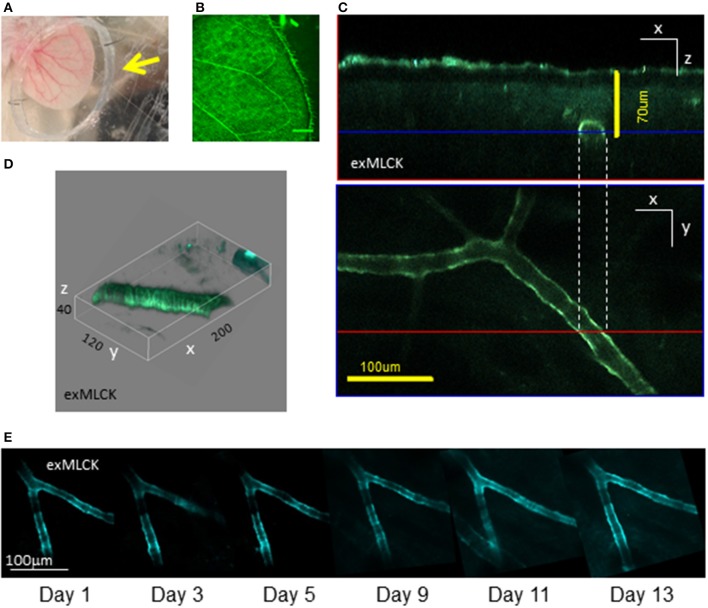
**Preparation of exMLCK mouse ear vasculature for *in vivo*, longitudinal, 2-photon imaging. (A)** Dorsal surface of the depilated ear of an anesthetized mouse. The ear is positioned within a recording chamber constructed of a polypropylene ring (arrowhead) pinned to a silicone block. Veins are readily visible and provide clear landmarks for repeated imaging. The ear is adhered via adhesive tape, pinned to a silicone block. The polypropylene ring retains water for the 20× dipping objective lens and has a cut-out region on its lower surface to ensure that blood flow is not occluded. The ear is thus positioned securely without any perturbation. **(B)** Confocal image (single photon excitation of YFP, YFP emission; bar = 800 μm) of the ear vasculature reveals the architecture of arteries and arterioles. Such low magnification images are used to precisely map the location of arterial sections used for longitudinal measurements. **(C)** Multiphoton excitation of CFP allows for FRET imaging of arterioles beneath light scattering tissue acquisition of multiple arterial sections (Z stack) enabling orthogonal view reconstruction. The blue and red lines denote the optical section shown in the paired panels. **(D)** 3D representation of a separate arteriole, *in vivo*, demonstrates the resolution of single smooth muscle cells (numbers denote axis scale in μm). **(E)** CFP channel images of the same arteriolar branch, ~18 μm in diameter, acquired on 6 separate occasions in a 13 day period. Scale bar = 100 μm. The data demonstrate the ability to locate particular sites in an individual animal, as required for longitudinal studies (reproduced with permission from AJP, Heart and Circulatory Physiology).

Surface hair on the ear was carefully depilated (Nair; Church & Dwight Co., Inc., Princeton, NJ) at least 3 days prior to imaging. Mice were lightly anesthetized (1.5% isoflurane) and then placed in a prone position on a temperature-controlled platform set to maintain core temperature at 37°C, which was monitored via rectal thermoprobe. The ventral surface of the depilated ear was affixed to a silicone platform with adhesive tape, situating the dorsal surface of the ear for imaging (Figure 2). This arrangement immobilized the ear, without directly pinning it, and provided a convenient configuration for use of a dipping objective lens (20×, 1.0 NA). Mice were taken off of isoflurane and allowed to regain consciousness after being placed inside the darkened imaging platform of an upright microscope. Telemetric blood pressure measurements distinguished the anesthetized and unanesthetized/awake conditions since isoflurane dependably lowers MAP (Figure 1). Moreover, transient fluctuations in the blood pressure waveform [associated with attempted movement of an animal (~3–4 per minute)] were typically absent in the anesthetized state.

### Fluorescence recording

A Zeiss LSM 710 NLO microscope was utilized for two-photon microscopy, equipped with a femtosecond pulsed near infrared (IR) laser (Chameleon Vision, Coherent, Inc., Santa Clara, CA). The frame rate and pixel size of the bidirectional scanning varied for each experiment, but ranged from 2 to 5 Hz and 0.59 to 0.83 μm/pixel, respectively. The microscope was enclosed in a light-tight enclosure and room lights were turned off to eliminate background signals. The CFP moiety of exMLCK was excitation at 820 nm. Emission light received IR filtration before being separated into two channels (CFP and YFP). CFP and YFP emission was band pass filtered from 460 to 500 nm and 520 to 560 nm, respectively. Two binary GaAsP photodetectors within the Zeiss LSM BiG module detected fluorescence emission. The gains of the two detector channels were held constant for all experiments at levels optimizing exMLCK fluorescence. The configuration settings of the detectors were reproduced during calibration experiments, which determined the maximum and minimum FRET ratios obtainable from our microscope (Mauban et al., 2014).

The theory of the use of the exMLCK FRET ratio under these conditions has been discussed extensively in our previous publications (Wier et al., 2008; Raina et al., 2009; Zhang et al., 2010; Wang et al., 2013). Briefly, the fractional occupancy of exMLCK by Ca^2+^/Calmodulin (Y) was calculated as Y = (R – R_min_)/(R_max_ – R_min_). The Calcium concentration wherein *Y* = 0.5 represents the EC_50_. The fluorescence ratios R_max_ and R_min_, respectively, represent 100 and 0% fractional occupancy of exMLCK by Ca^2+^/Calmodulin. Free [Ca^2+^] is calculated from Y, using the Hill equation, as [Ca^2+^] = [(Y·EC^n^_50_)/(1.0 – Y)]^1/n^. The relationship between free [Ca^2+^] and exMLCK FRET ratio, *R*, as measured in α-toxin permeabilized mesenteric small arteries, is well-fitted by the Hill equation, with an EC_50_ (K_A_) of 0.892 μM (pCa, 6.05) and a Hill Coefficient (*n*) of 1.4 (Wang et al., 2013). Methods for determination of intrinsic fluorescence, spectral overlap and other considerations for quantitative imaging of [Ca^2+^] using exMLCK FRET ratio measurements under these conditions were described previously (Wier et al., 2008; Zhang et al., 2010; Mauban et al., 2014).

### Intraperitoneal (i.p.) injections

A 32 mm, 21 gauge flexible catheter (Abbott Laboratories, Abbott Park, IL) was inserted in the abdominal cavity and anchored to the skin with adhesive tape to intraperitoneally deliver hexamethonium 30 μg/g BW (Sigma-Aldrich, St. Louis, MO) dissolved in sterile saline (0.9% NaCl).

### Data analysis and statistics

Data are expressed as means ± s.e.m.; *n* denotes the number of animals. Comparisons of data were made using Student's paired or unpaired *t*-test, as appropriate. Differences were considered significant at *P* < 0.05. Image processing was via custom software routines using IDL (Exelisvis, CO). Blood pressure measurements were analyzed with DSI software.

## Results

### Effects of conscious restraint on MAP

**Figure 3:**
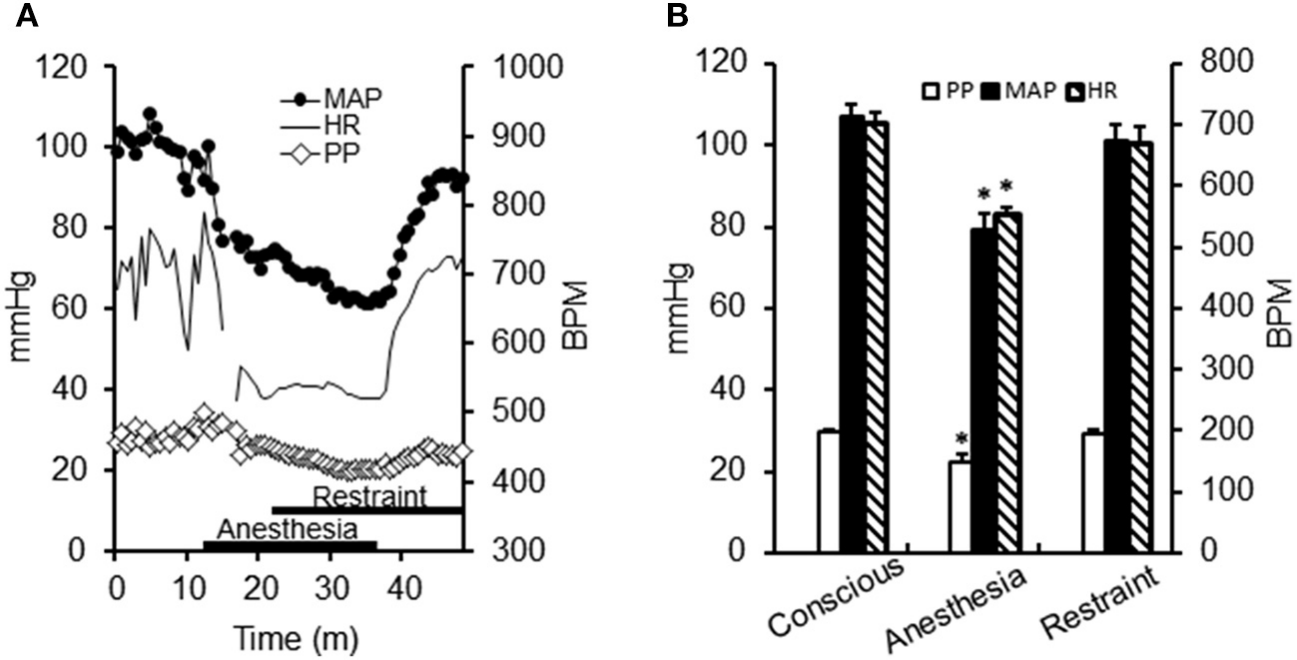
**Effect of anesthesia and conscious restraint on heart rate and blood pressure. (A)** Telemetric recordings of one mouse [Mean arterial pressure (MAP), closed circles; heart rate (HR), solid line; pulse pressure (PP), open diamonds] during preparation for ear arteriole imaging. Recording began under freely mobile conditions, after which the animal was restrained under a brief period of isoflurane anesthesia (1.5%). Anesthesia depressed all variables, which were restored upon removal of anesthesia. **(B)** Summary data (*n* = 4) demonstrate that all variables significantly decreased during isoflurane anesthesia (^*^P < 0.05) and were unaltered during experimental restraint.

Mice typically required 7–10 days to regain normal MAP following surgical implantation of telemetric blood pressure transducers and head restraints. MAP recordings began as the animal was engaging in normal behavior within its housing cage. Recordings continued into isoflurane induction while the animal was affixed to the restraint device and also during restoration of consciousness (upon removal of isoflurane) while restrained (Figure 3). Because two-photon fluorescence imaging was being used, the immediate environment of the animal was dark, a factor which may have influenced the MAP in the conscious restrained state. After awakening in the dark, MAP in the conscious restrained animal (101 ± 4 mmHg, *n* = 4) was not significantly different from that in the conscious, unrestrained animal (107 ± 3 mmHg) (Figure 3A). In contrast, 1.5% isoflurane significantly lowered MAP (79 ± 4 mmHg, *P* < 0.05). Mice periodically attempted activity ~3–4 times per minute, and this was associated with transient increases in MAP, as occurs in the normal basal state.

### Ca^2+^ signaling

**Figure 4:**
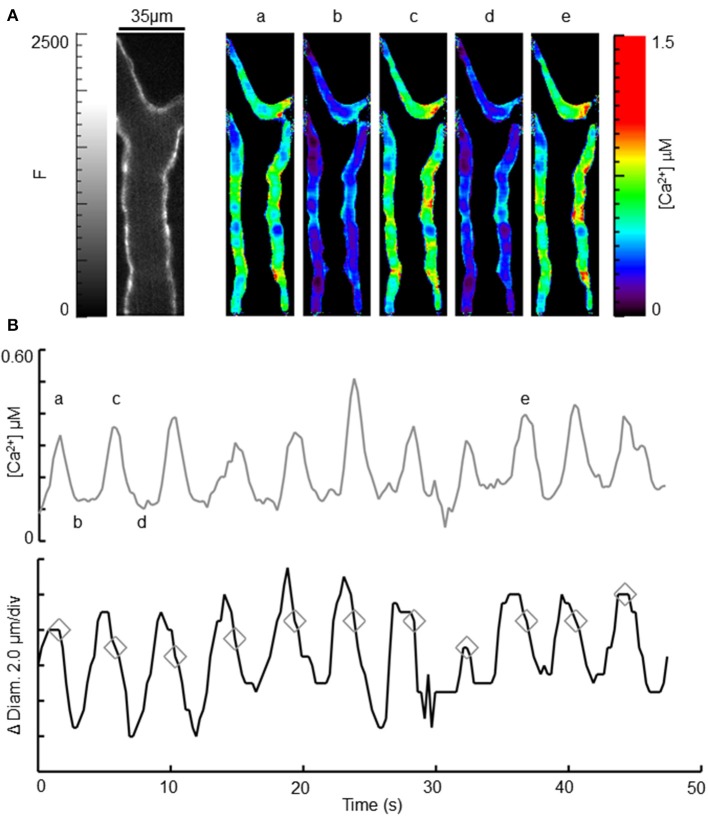
**Observation of intracellular [Ca^2+^] and diameter during oscillatory vasomotion. (A)** Left image shows an ear arteriole using CFP fluorescence (0–2500). Scale bar is 35 μm and applies to all images. White triangles denote the 5 ROIs used to measure [Ca^2+^]. Rainbow-colored images to the right demonstrate the range of calculated [Ca^2+^] (0–1.5 μM) over the time-course of spontaneous oscillatory vasomotion, as indicated by the small letters above each image, relating to **(B)**. **(B)** Average [Ca^2+^] and change in wall position (change in diameter) from the 5 ROIs labeled in **(A)**. Peak oscillations in [Ca^2+^] are indicated by gray diamonds in the diameter trace, and correspond with the rapid decrease in diameter during vasomotion. See Supplemental Video.

**Figure 5:**
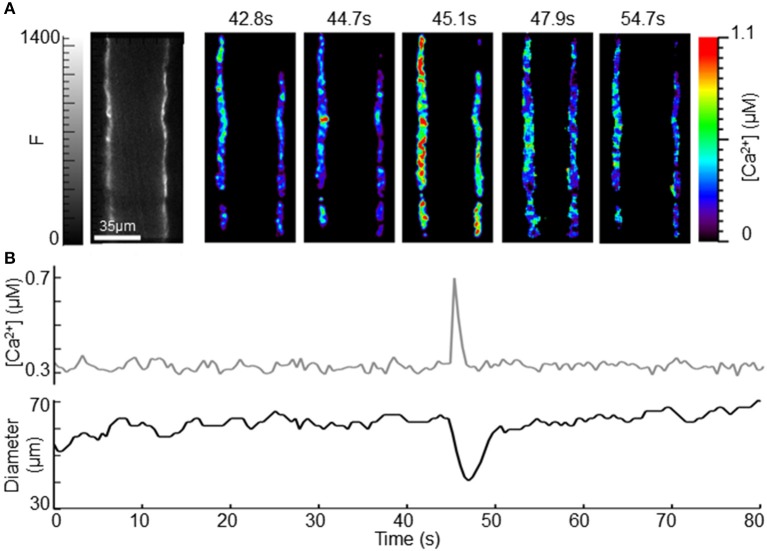
**Observation of intracellular [Ca^2+^] and diameter during a spontaneous vasoconstriction. (A)** CFP channel fluorescence, calculated [ca^2+^], **(B)** mean [Ca^2+^] from 5 ROIs and diameter in an exMLCK ear arteriole during 80 s of conscious restraint. Imaging scales are presented in grayscale to show CFP fluorescence (0–1400) and rainbow to show calculated [Ca^2+^] (0–1.1 μM). The 5 rainbow images show the frames immediately surrounding the transient increase in [Ca^2+^] occurring at 45.1 s. Vasoconstriction occurred in the frame following the peak Ca^2+^ and then immediately returned to basal diameter.

Oscillatory vasomotion and accompanying regular oscillations in [Ca^2+^] were present in 71% (*n* = 7) of the arterioles observed in the conscious, restrained state (Figure 4). Changes in [Ca^2+^] were nearly uniform and synchronous between smooth muscle cells. Such synchronous Ca^2+^ oscillations were not reported previously in any of the arterioles examined in anesthetized biosensor mice (Mauban et al., 2014) and were less frequent during anesthetized measurements in this study 14% (*n* = 7). For the arteriole illustrated in Figure 4, the time-averaged diameter was about ~35 μm and the oscillatory vasomotion involved wall movements of ~5–6 μm and [Ca^2+^] oscillations from ~0.1 to ~0.5 μM (Figure 4B and Supplemental Video). Maximum [Ca^2+^] occurred during the rapid decrease in diameter, as would be expected for a Ca^2+^ indicator that binds Ca^2+^ with similar affinity to the endogenous MLCK and which activate cross-bridge cycling (Hong et al., 2009). In some instances, very rapid and brief Ca^2+^ transients were observed (Figure 5) and these were accompanied by similarly rapid and brief vasoconstriction. In this case, the Ca^2+^ transient peaked before contraction was observed; contraction was evident in the subsequent frame. As with the oscillatory vasomotion, the changes in [Ca^2+^] and wall motion appeared synchronized throughout the arterial wall.

**Figure 6:**
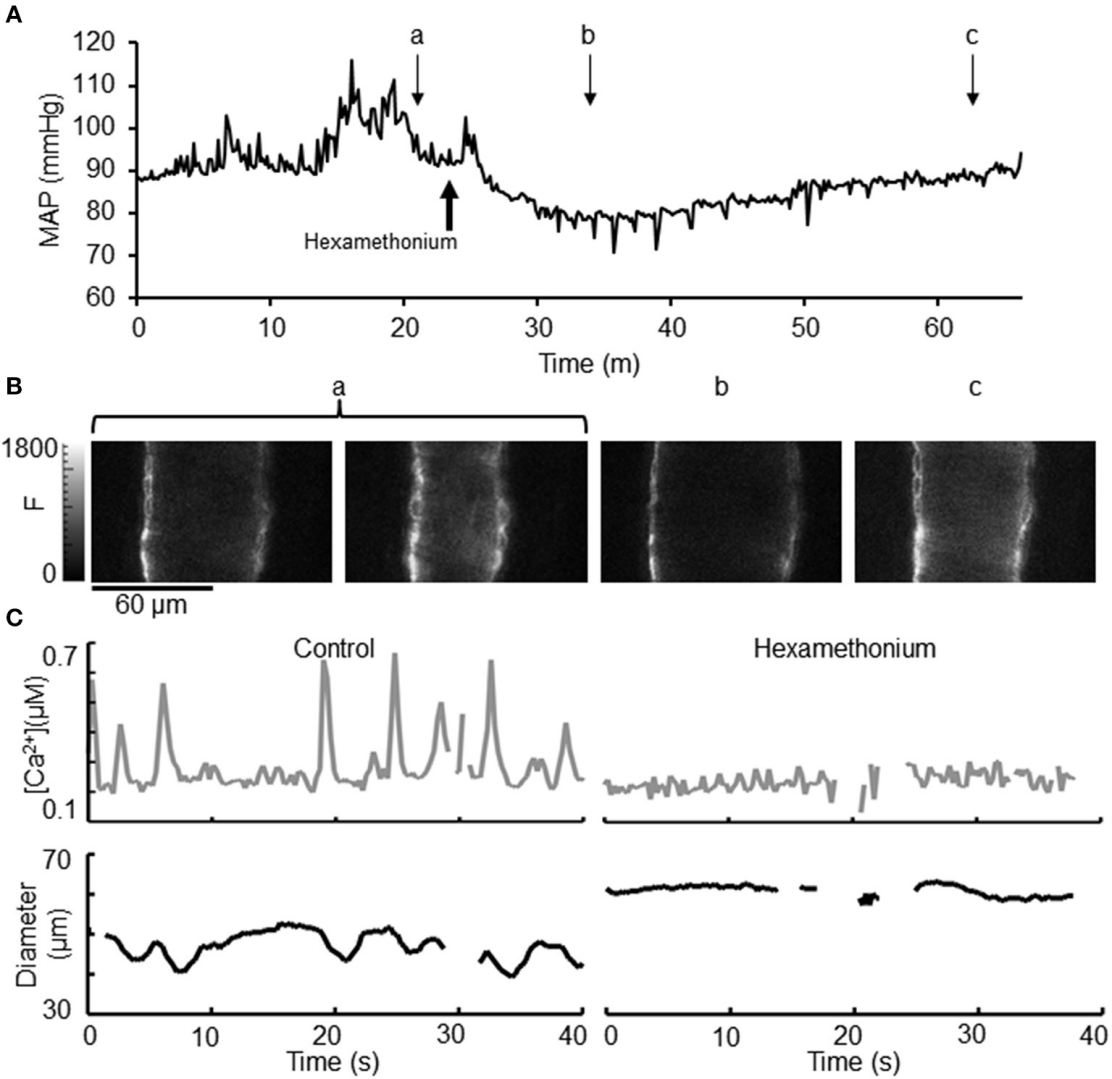
**Effect of hexamethonium on [Ca^2+^] oscillations, vasomotion, and diameter in exMLCK ear arterioles. (A)** Mean arterial pressure (MAP) before, during and following recovery of i.p. hexamethonium injection (30 μg/g BW). Brief attempts of activity by the mouse are indicated by transient changes in MAP. Small letters indicate the time point of the corresponding CFP images shown in **(B)**. **(B)** Selected images demonstrate that arteriolar vasomotion **(a)** is eliminated by hexamethonium treatment **(b)**. Recovery of tone **(c)** occurred ~30 min after hexamethonium injection. **(C)** Transient increases in [Ca^2+^] and the corresponding decreases in diameter during irregular vasomotion during conscious, restrained conditions (left). Hexamethonium eliminated vasomotion, [Ca^2+^] transients, and caused vasodilation (right). Baseline [Ca^2+^] was not affected by hexamethonium.

As we had hypothesized that recordings in the conscious state would preserve normal SNA and MAP, we examined the effect of autonomic ganglionic blockade on MAP, arteriolar dimensions and Ca^2+^ signaling (Figure 6). Intra-peritoneal (i.p.) injection of hexamethonium (30 μg/g BW) (which transiently blocks all SNA in anesthetized mice), reduced MAP within a few minutes of injection, reaching a minimum 78 ± 4 mmHg (Figure 6Ab). An identified small artery was undergoing vasomotion (Figure 6Ba), however during the minimum MAP following hexamethonium treatment (Figure 6Ab), no vasomotion was present in this artery (Figure 6Bb) and the diameter was significantly increased. During recovery, MAP slowly increased (Figure 6Bc) and this artery regained some of its vasomotion behavior and initial tone. In a separate experimental animal, hexamethonium treatment was also seen to abolish the Ca^2+^ signaling associated with vasomotion (Figure 6C).

**Figure 7:**
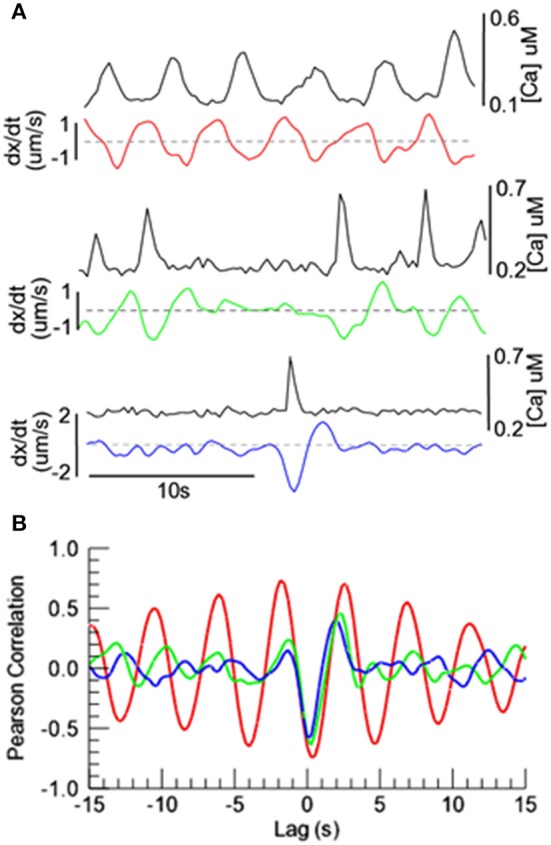
**Temporal relationships between [Ca^2+^] oscillations and diameter in exMLCK ear arterioles. (A)** Vasomotor activity from Figures 4b, 5, 6c, illustrating [Ca^2+^] and the rate of change in diameter for regular and irregular oscillatory vasomotion, and a spontaneous vasoconstriction. Black traces represent [Ca^2+^] and colored traces represent the time derivative (dx/dt) of arterial diameter. Decreasing arterial diameter produces negative derivative values. **(B)** Cross correlation of [Ca^2+^] and dx/dt. The strongest correlation between [Ca^2+^] and dx/dt occurs at a lag of ~0.3 s, indicating that the maximum rate of vasoconstriction follows the peak [Ca^2+^] with a delay of ~0.3 s.

Whether the oscillating vasomotion was highly regular (Figure 4), irregular (Figure 6), or was an isolated instance of vasoconstriction (Figure 5), the peak of the [Ca^2+^] appeared to coincide approximately with the maximum rate of vasoconstriction. In order to examine this possible correlation quantitatively, we obtained the cross-correlation between the [Ca^2+^] and the time derivative (dx/dt) of arterial diameter for these three types of vasomotion (Figure 7). In all cases, the correlation between [Ca^2+^] and the rate of diameter change was strongest at a lag of ~0.3 s. (At this lag, Pearson's correlation is negative because high [Ca^2+^] is associated with a decrease change in diameter; a negative time derivative). Thus, peak [Ca^2+^] is immediately followed by the maximum rate of vasoconstriction. With greater positive and negative lags, strong correlations occurred only with regular oscillating vasomotion, as would be expected for repetitive, stereotypical events. Irregular oscillating vasomotion and spontaneous vasoconstrictions exhibited no additional strong correlations other than the primary 0.3 s lag.

## Discussion

The major advantage of making [Ca^2+^] measurements in a conscious animal (Wier, 2014) should be that arterioles are in a state closer to physiological than can be achieved in any other condition. Our expectation is that cardiovascular control systems, autonomic nerve activity, endothelial, hormonal, and myogenic mechanisms should be operating similarly to the animal's normal basal state under the conditions of our imaging experiments. The primary indication of this is MAP at normal basal levels, rather than being altered. MAP in the conscious restrained mice, set up for two-photon imaging of ear arterioles, was not different from that when the animal was moving freely before the experiment began. Furthermore, MAP fluctuates during imaging, as it does normally. Such fluctuations in MAP in the freely moving animal are associated with activity, and in the conscious restrained state, with attempted activity (as evidenced by slight movements that were not fully prevented). With respect to MAP, our results are somewhat different than those reported earlier (Gross and Luft, 2003) where restraint of mice did result in significant elevation of MAP. In those experiments, mice were not restrained in the dark, and were under a different system of restraint. Whatever the reason for the differences, the key result for cardiovascular experiments of the type reported here is that both MAP and HR were not altered by stress during the system of conscious restraint that we used. Therefore, studies of experimental hypertension or investigations of mechanisms of normal vascular control may not be complicated by stress-induced or anesthesia-induced alterations in SNA or hormonal control systems. Blood flow and arterial pressure are maintained so that endothelial flow-sensitive mechanisms and pressure sensitive mechanisms (myogenic tone) should be operating normally.

Arterioles undergoing oscillatory vasomotion (Figure 4) were found much more frequently in conscious mice than in anesthetized mice (in agreement with an earlier study; Drew et al., 2011), and such vasomotion was strongly reduced or abolished by inhibition of SNA (Figure 6) by hexamethonium. With respect to Ca^2+^ dynamics, diameter changes and frequency, the vasomotion appeared similar to that elicited by exposure of isolated arteries to adrenergic receptor agonists, such as phenylephrine (Mauban et al., 2001). Furthermore, cross correlation results suggested broad similarity in Ca^2+^-mediated vasoconstriction among the vasomotion and spontaneous vasoconstriction observed during consciousness. Thus, it seems likely that the vasomotion we observed was due to the action of the sympathetic neurotransmitter, norepinephrine (NE), acting on α_1_-adrenergic receptors (α_1_-AR) (Zacharia et al., 2013). The cellular mechanisms of adrenergic vasomotion remain unclear, but likely involve a voltage-dependent synchronization of sarcoplasmic reticulum Ca^2+^ release and Ca^2+^ entry through voltage dependent Ca^2+^ channels (Aalkjaer et al., 2011). The fact that volatile anesthetics such as Isoflurane induce membrane hyperpolarization and vasodilation (Yamazaki et al., 1998), likely explains at least part of the mechanism for reduced vasomotion relative to the conscious state.

In isolated arteries, stimulation of sympathetic nerve terminals within the arterial wall elicits several types of Ca^2+^ signals (Wier et al., 2009). Neurally-released ATP activates P2X1 receptors to produce a local Ca^2+^ transient, termed “jCaT” or junctional Ca^2+^ transient (Lamont and Wier, 2002). JCaTs are too fast and small to be detectable with exMLCK in ear arterioles, and the optical resolution is probably inadequate. We do hypothesize that the Ca^2+^ flashes we observed are the result of a synchronous smooth muscle action potential, triggered by excitatory junction potentials (EJPs) activated by neurally released ATP. Such Ca^2+^ transients may thus reflect mainly Ca^2+^ entry through voltage-gated Ca^2+^ channels. Thus, smooth muscle membrane potential appears to be critical to control of contraction of these arterioles *in vivo*.

Clearly, highly synchronized Ca^2+^ signaling drives synchronous contractile activation in arterioles in conscious animals. Although the physiological significance of such vasomotion remains unclear, it is thought likely to enhance tissue dialysis (for review, see Aalkjaer et al., 2011). Interestingly, NE induces vasomotion more readily in mesenteric small arteries from spontaneously hypertensive rats (SHR) than in normotensive rats (Chen et al., 2010).

In isolated pressurized arteries, asynchronous propagating Ca^2+^ waves are readily produced by exposure to α_1_-AR agonists. It has also been suggested that such Ca^2+^ waves are involved in myogenic tone (Mufti et al., 2010). However, asynchronous propagating Ca^2+^ waves were not seen in any blood vessel in the present study. Such Ca^2+^ signals were also not detected in our previous studies, either in cremaster muscle arterioles (Mauban et al., 2013) or femoral arteries (Wang et al., 2013; Zacharia et al., 2013) that were surgically exposed, or in intact ear arterioles (Mauban et al., 2014). Thus, it appears that the Ca^2+^ waves observed *in vitro* remain elementary signals and fail to emerge as a phenomenon in a fully intact physiological system.

In summary, high resolution imaging of Ca^2+^ signaling in the smooth muscle cells of blood vessels in the ears of restrained conscious mice is readily achieved through the use of optical biosensor mice and two-photon microscopy. Arterioles and small arteries of the mouse ear are under tonic control by the sympathetic nervous system, and the predominant mode of contractile activation seems to be spatially uniform Ca^2+^ signaling, insofar as can be observed with exMLCK. Although both myogenic tone, and sympathetic neurogenic contractions of isolated arteries can involve asynchronous propagating Ca^2+^ waves in individual cells, such Ca^2+^ signals were never observed in the present study. These results reinforce the notion that synchronization of Ca^2+^ signaling *in vivo* is important for physiological function.

### Conflict of interest statement

The authors declare that the research was conducted in the absence of any commercial or financial relationships that could be construed as a potential conflict of interest.
